# Correlation Between Hippocampal Enlarged Perivascular Spaces and Cognition in Non-dementic Elderly Population

**DOI:** 10.3389/fneur.2020.542511

**Published:** 2020-09-29

**Authors:** Jae Eun Sim, Moo-Seok Park, Hee-Young Shin, Hyun-Soon Jang, Hong-Hee Won, Sang Won Seo, Woo-Keun Seo, Byoung Joon Kim, Gyeong-Moon Kim

**Affiliations:** ^1^Department of Neurology, Geumcheon Su Hospital, Seoul, South Korea; ^2^Department of Neurology, Seoul Medical Center, Seoul, South Korea; ^3^Department of Health Promotion Center, Samsung Medical Center, Sungkyunkwan University School of Medicine, Seoul, South Korea; ^4^Department of Neurology, Anseong St. Mary Hospital, Anseong, South Korea; ^5^Department of Health Sciences and Technology, Sungkyunkwan University School of Medicine, Suwon-si, South Korea; ^6^Department of Neurology, Samsung Medical Center, Sungkyunkwan University School of Medicine, Seoul, South Korea

**Keywords:** hippocampal perivascular enlarged space, pulsatility index, Alzheimer's disease assessment scale-cognitive subscale (ADAS-cog), memory, medial temporal atrophy

## Abstract

**Background and aims:** The pathophysiology of hippocampal enlarged perivascular spaces (H-EPVS) and its relationship to cognitive impairment is largely unknown. This study aimed to investigate the relationship between H-EPVS and cognition in non-dementic elderly population.

**Methods:** A total of 109 subjects were prospectively enrolled. The eligibilities for inclusion were age from 55 to 85 years and Mini-Mental Status Examination score of ≥26. The Alzheimer's Disease Assessment Scale-Cognitive Subscale (ADAS-Cog), Montreal Cognitive Assessment, transcranial Doppler (TCD), and brain magnetic resonance imaging results were evaluated. H-EPVS was categorized in a three-degree scale: degree 0 (no), degree 1 (1,2), and degree 2 (>2). The associations between H-EPVS and TCD parameters/cognitive test profiles were analyzed.

**Results:** The mean age was 65.2 years, and 52.3% subjects were men. H-EPVS was found to be associated with age (degree 2 vs. degree 1 vs. degree 0, 69.20 ± 6.93 vs. 65.70 ± 5.75 vs. 63.80 ± 5.43; *p* = 0.030) and ADAS-Cog memory score (degree 2 vs. degree 1 vs. degree 0, 14.88 ± 4.27 vs. 12.49 ± 4.56 vs. 11.4 ± 4.23; *p* = 0.037). However, the pulsatility index was not related to the degree of H-EPVS. Multivariate analysis revealed medial temporal atrophy (MTA) scale score was independently associated with ADAS-Cog memory score (MTA scale sum ≥4, *p* = 0.011) but not with the degree of H-EPVS. MTA scale score showed correlation with H-EPVS (*r* = 0.273, *p* = 0.004).

**Conclusions:** Aging was associated with the development of H-EPVS in non-dementic elderly population. Memory function was found to be associated with MTA but not with the degree of H-EPVS.

## Introduction

The majority of patients who developed dementia had a smaller baseline hippocampal volume before their clinical diagnosis than individuals who remained free from dementia (1). While hippocampal volumes have been extensively examined in patients with cognitive impairment, signal variation within the hippocampus commonly observed on magnetic resonance imaging (MRI), described as hippocampal enlarged perivascular spaces (H-EPVS), have received less attention. Various radiological terminologies have been previously used for this feature (2, 3). Recently, it has been called an enlarged perivascular space (4–6). The prevalence of hippocampal enlarged perivascular spaces (H-EPVS) seems to about more than half in healthy elderly populations (2–4). However, the risk factors of H-EPVS, their radiological relevance, and their impact on cognitive performance remain under-investigated until now.

The severity of basal ganglia enlarged perivascular spaces (BG-EPVS) or white matter enlarged perivascular spaces (WM-EPVS) was recently found to be associated with an increased risk of cognitive decline or incident dementia (7). The BG-EPVS is more strongly linked to markers of small vessel disease in patients with stroke and hypertensive vasculopathy in patients with cognitive impairment (1, 8–10). The WM-EPVS was associated with incident dementia in healthy subjects (11). Centrum semi-ovale EPVS severity is strongly associated with clinically diagnosed Alzheimer's disease (AD)-related cognitive impairment (12). However, the association between the degree of H-EPVS and cognitive impairment is controversial. Previous studies have investigated the effect of H-EPVS on cognition, but the results were inconsistent. Considering H-EPVS, the results are not associated with cognitive ability or development of dementia (2, 3, 13). Higher H-EPVS counts were linked to better cognitive performance in memory (5). There are fewer studies on the relationship of H-EPVS and cognitive impairment. H-EPVS was associated with global cognitive function measured using the Mini-Mental State Examination (MMSE) (14) and worse verbal reasoning function (4). Further investigation of H-EPVS and cognition in larger cohorts without cerebrovascular and amyloid burden (e.g., healthy elderly cohorts) will help clarify the correlation of H-EPVS and cognition.

It has been suggested that EPVS is a pathologic hallmark of compromised fluid and toxin clearance from the brain and a potential biomarker of neurovascular and neurodegenerative diseases (15). Parenchymal amyloid β could reach the vessel walls through perivascular spaces, eventually aggregating within them and causing retrograde perivascular enlargement due to interstitial space fluid blockage (10, 16). The vascular pathology causes a decrease in vessel wall compliance, increaseing cerebrovascular pulsatility. The change in cerebrovascular pulsatility affects glymphatic homeostasis and brain clearance (10, 17). Impaired vascular circulation from any cause leads to cognitive impairment (18, 19). The middle cerebral artery-pulsatility index (MCA-PI) is independently associated with cognitive impairment in middle-aged asymptomatic subjects (20). Patients with AD with higher posterior atrophy scores have worse performance on tasks of visuospatial and executive function (21).

We hypothesized that the degree of H-EPVS is related to increased arterial PI and cognitive impairment. This study aimed to investigate risk factors of H-EPVS and the relationship between H-EPVS and PI or cognition particularly memory domain in non-dementic elderly population.

## Materials and Methods

### Subjects

Subjects were prospectively recruited from the outpatients who underwent brain MRI and transcranial Doppler (TCD) in the Samsung Medical Center health promotion center between October 2015 and August 2017. Then subjects were clinically evaluated and underwent cognition test, The Alzheimer's Disease Assessment Scale-Cognitive Subscale (ADAS-Cog), MMSE, and Montreal Cognitive Assessment (MoCA). The eligibilities for inclusion were age from 55 to 85 years and MMSE score of ≥26. Individuals with evidence of dementia, structural brain alternation including mass, stroke, and psychiatric problems were excluded. The complete study protocol was approved by the Ethical Committee of the Sungkyunkwan University. All subjects provided written informed consent. The following clinical variables were recorded for each subject: age, sex, education, smoking, alcohol consumption, and presence of hypertension, diabetes, and hypercholesterolemia. Education was defined as years of formal education. Smoking habit was categorized as current smoker or ex-smoker/never smoker. Hypertension was defined as previous diagnosis of hypertension (>140/90 mmHg) or use of antihypertensive treatment for control of blood pressure. Diabetes was defined as previous diagnosis of diabetes or current use of antidiabetic drugs. Hypercholesterolemia was defined as previous diagnosis of hypercholesterolemia or current use of antihyperlipidemic medications. We examined the blood for urea and electrolyte level, calcium level, liver function, glucose level, hemoglobin level, white cell count, platelet count, and cholesterol level.

### Image Acquisition

Each patient underwent 3-Tesla MRI (Achieva; Philips Medical System, Best, the Netherlands) including diffusion-weighted imaging, conventional T1- and T2-weighted imaging, T2 fluid attenuated inversion recovery imaging, gradient echo imaging, 3D time-of-flight magnetic resonance angiography (MRA), and contrast enhanced MRA. Three-Tesla MRI (Achieva; Philips Medical System, Best, the Netherlands) was used to acquire 3D T1 turbo field echo MRI data with the following imaging parameters: sagittal slice thickness, 1.0 mm; over contiguous slices with 50% overlap; no gap; repetition time, 9.9 ms; echo time, 4.6 ms; flip angle, 8°; and matrix size of 240 × 240 pixels reconstructed to 480 × 480 over a field of view of 240 mm.

### TCD Protocol

TCD (Pioneer TC 8080; Nicolet Vascular, Madison, WI, USA) was used to monitor both MCAs with insonation depths of 40–60 mm and both posterior cerebral arteries (PCAs) with insonation depths of 60–70 mm. We followed the TCD criteria based on consensus criteria of the fourth meeting of the European Society of Neurosonology and Cerebral Hemodynamics. The TCD recording quality was continuously observed by one investigator. Data were automatically saved on the computer hard disk for review, and all analyses were performed blinded to the individual patient details.

### Rating of H-EPVS

H-EPVS were defined as CSF-like signal-intensity change (hypointense on T1 and hyperintense on T2) that were round, curvilinear, or crescent, <3 mm in their maximum diameter (22), with smooth delineated contours, and typically located in the lateral portion of the hippocampus (2). H-EPVS were identified on T1 coronal images. For lesions fulfilling the abovementioned criteria but having maximum diameter ≥3 mm, only those with a typical vascular shape and following the orientation of perforating arteries were diagnosed (11).

**Figure 1:**
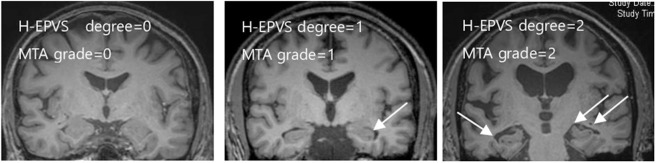
Examples of the degree of H-EPVS and MTA scale score. The three MRI images include each degree of H-EPVS and MTA scale score. White arrows point examples of H-EPVS. H-EPVS, hippocampal enlarged perivascular spaces; MTA, medial temporal atrophy.

H-EPVS were individually and separately scored on the right and left side of the hippocampus by two experienced neurologists who were blinded to all clinical data. Two independent raters blinded to each other's readings rated H-EPVS manually. In case of disagreement, a consensus meeting was held. The sum of bilateral sides was used later for statistical analysis. We categorized in a three-degree scale (3) according to the sum of H-EPVS in the left and right hippocampus: degree 0 (no), degree 1 (1,2), and degree 2 (>2). Intra- and inter-observer agreement was assessed using a weighted Cohen κ statistics. κ-value was 0.759. Figure 1 demonstrates H-EPVS example of each severity degree of both hippocampi.

### Hippocampal Atrophy

Semiquantitative analysis of hippocampal atrophy by medial temporal atrophy (MTA) scale score was administered. The image analysis included a visual rating of MTA on the T1-weighted images. T1-weighted images were viewed in the coronal plane, and MTA scale scores for the left and right hemispheres were provided. MTA was rated on a 5-point scale (0 point, absent; 1 point, minimal; 2 points, mild; 3 points, moderate; and 4 points, severe) based on the height of the hippocampal formation and width of the choroid fissure and temporal horn. The sum of the MTA scale scores from both hemispheres were calculated for the statistical analysis. Quantitative analysis of hippocampal volume was estimated using Inbrain® software (https://www.inbrain.co.kr/) (23, 24). Based on FreeSurfer 6.0, the hippocampal volume values of Inbrain® software are the same. The sum of both hippocampal volumes were calculated for the statistical analysis.

### Cognitive Assessment

Cognitive function was assessed using the ADAS-Cog test that included several domains. All tests were conducted by the same tester who was blinded to the results of brain imaging and biochemistry. ADAS-Cog is the most widely used general cognitive measure in clinical trials of AD. We divided into subgroups by cognitive domain (25, 26). For the memory domain including an orientation part, we checked word recall of 12 items, word delayed recall, and word recognition. For the language function, we checked naming, remembering test instructions, spoken language ability, word finding difficulty, and comprehension. For praxia, we checked commands, constructional praxia, and ideational praxia. A higher ADAS-Cog score reflected lower performance. We also performed MMSE and MoCA to assess global cognition. Finally, the geriatric depression scale was administered to assess the presence of depression.

### Statistical Analysis

Initially, we divided the H-EPVS count and categorized it into three degrees. To study the risk factors associated with H-EPVS, we performed appropriate univariate tests to compare clinical and radiologic data between subjects with high and low degrees of H-EPVS. Continuous values are adjusted to mean ± standard deviation. The ANOVA and Kruskal Wallis test were applied for continuous variables, while the χ^2^-test and Fisher's exact test were applied to categorical variables. After we found that ADAS-Cog memory score was related to the degree of H-EPVS, particularly degree 2, we divided subjects into two groups by ADAS-Cog memory to study the risk factors associated with memory cognitive function. The *t*-test and Wilcoxon rank sum test were applied to continuous variables, while the χ^2^-test and Fisher's exact test were applied to categorical variables. The association of memory cognitive function and degree of H-EPVS, MTA scale sum score and hippocampal total volume was determined using linear regression to obtain effect estimates and standard errors. Then, adjustments for covariates were constructed initially for age, sex, education, high-density lipoprotein (HDL), and hypertension, which are variables showing differences in the univariate analysis (*p* < 0.1) and subsequently for degree of H-EPVS, MTA scale sum score and hippocampal total volume. We used the R software (R version 3.6.5, The R Foundation for Statistical Computing). Two-tailed *p* < 0.05 were considered statistically significant.

## Results

### Baseline Characteristics

In a 2-year period, 109 subjects were enrolled. The overall demographics, clinical characteristics, blood chemistry results, and H-EPVS count are presented in Table 1. The mean age of subjects was 65.2 years (SD = 5.94), and 52.3 % (*n* = 57) subjects were men. Sixty-two (56.9 %) individuals had at least 1 H-EPVS with a maximum number of 5. Degree 1 was the most frequent H-EPVS degree, accounting for 45.9% of cases. Figure 1 shows the representative MRI of each degree of H-EPVS.

**Table 1:** Baseline characteristics of the study subjects.

**Characteristics (*n* = 109)**	
Age (year) (range)	65.2 ± 5.94 (55–85)
Male sex, *n* (%)	57 (52.3)
Education (year)	12.4 ± 3.75
Hypertension, *n* (%)	46 (42.2)
Diabetes, *n* (%)	17 (15.6)
Hyperlipidemia, *n* (%)	50 (45.9)
Coronary artery disease, *n* (%)	12 (11)
Depression, *n* (%)	5 (4.6)
Current smoking, *n* (%)	11 (10.1)
Current alcohol consumption, *n* (%)	58 (53.2)
SBP (mmHg)	125 ± 16.0
DBP (mmHg)	72.8 ± 8.91
FBS (mg/dL)	102 ± 17.6
T-Chol (mg/dL)	186 ± 38.4
LDL (mg/dL)	122 ± 36.7
HDL (mg/dL)	55.1 ± 13.0
TG (mg/dL)	124 ± 61.8
Number of H-EPVS	
0	47
1–2	50
>2	12
GDS	2.73 ± 2.93
MMSE	28.5 ± 1.29
MoCA	25.9 ± 2.55
MoCA memory	2.64 ± 1.68
ADAS-Cog	15.2 ± 5.46
ADAS-Cog-language	0.339 ± 0.748
ADAS-Cog-memory	12.2 ± 4.45
ADAS-Cog-praxia	2.52 ± 1.60

*Key: SBP, systolic blood pressure; DBP, diastolic blood pressure; FBS, fasting blood sugar; T-chol, total cholesterol; LDL, low-density lipoprotein; HDL TG, triglyceride; H-EPVS, hippocampal enlarged perivascular spaces; GDS, geriatric depression scale; MMSE, Mini-Mental State Examination; MoCA, Montreal Cognitive Assessment; ADAS-Cog, The Alzheimer's Disease Assessment Scale-Cognitive Subscale*.

### Risk Factor of H-EPVS

**Figure 2:**
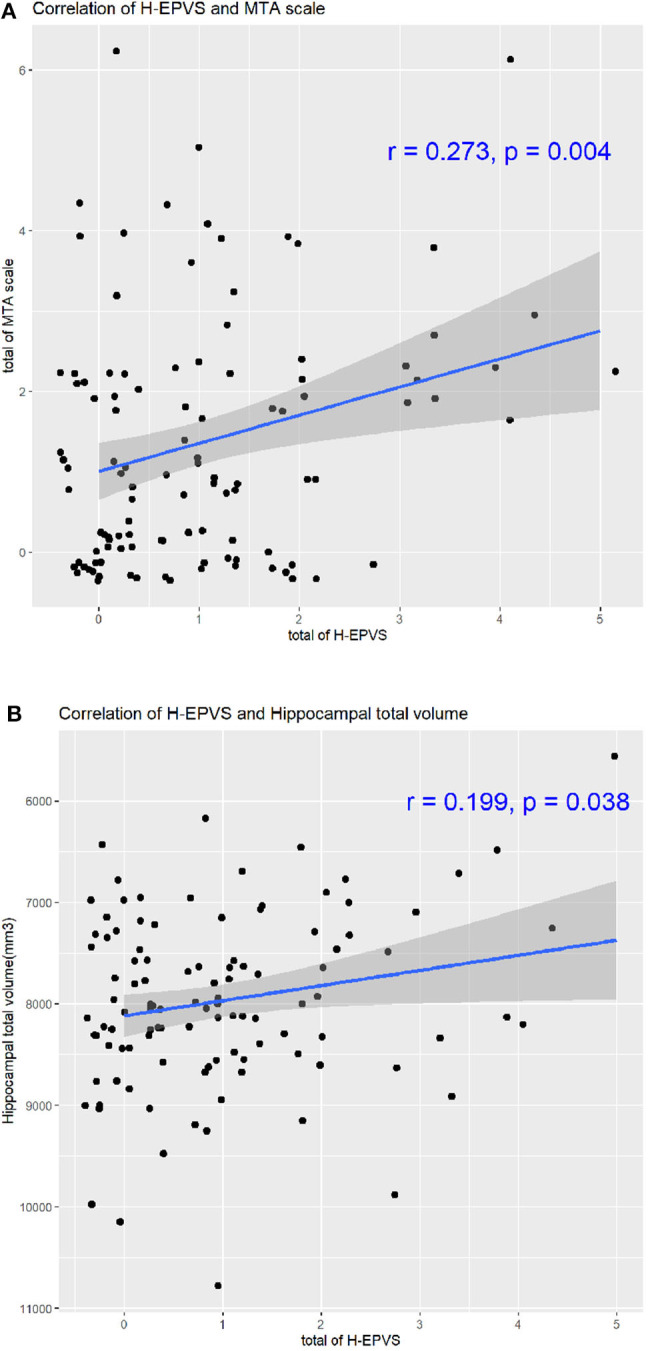
Correlation of H-EPVS and hippocampal atrophy. The line in each graph was obtained from the univariate regression model. R means correlation coefficients. Correlation of H-EPVS and MTA scale **(A)**. Correlation of H-EPVS and hippocampal total volume **(B)**. H-EPVS, hippocampal enlarged perivascular spaces; MTA, medial temporal atrophy.

The demographics, laboratory results, cognitive function, and brain imaging findings of the degree of H-EPVS are presented in Table 2. The degree of H-EPVS was associated with age (degree 2 vs. degree 1 vs. degree 0, 69.20 ± 6.93 vs. 65.70 ± 5.75 vs. 63.80 ± 5.43, *p* = 0.030). In the ordinary logistic regression analysis for association between H-EPVS and age, *P*-value was 0.006. Odds ratio was 1.095 and 95% confidence interval was 1.028–1.169. The degree of H-EPVS was related to ADAS-Cog memory score (degree 2 vs. degree 1 vs. degree 0: 14.88 ± 4.27 vs. 12.49 ± 4.56 vs. 11.40 ± 4.23, *p*-value 0.037). MTA scale sum score was increased with increasing degree of H-EPVS (degree 2 vs. degree 1 vs. degree 0, 2.50 ± 1.45 vs. 1.34 ± 1.44 vs. 1.06 ± 1.39; *p* = 0.005). H-EPVS and MTA scale score, hippocampal total volume showed the correlation (Figure 2, correlation efficient, 0.273; *p* = 0.004; correlation efficient, 0.199; *p* = 0.038). In contrast, the degree of H-EPVS had no association with sex, smoking, or alcohol drinking status, hypercholesterolemia, diabetes, coronary heart disease, and depression. *P*-value of current smoking was 0.094.

**Table 2:** Demographic, laboratory results, cognitive function, and brain imaging findings according to the degree of H-EPVS.

	**Degree 0 (*n* = 47)**	**Degree 1 (*n* = 50)**	**Degree 2 (*n* = 12)**	***P*-value**
Demography				
Male, *n* (%)	25 (53.2)	26 (52)	6 (50.0)	0.979
Age	63.80 ± 5.43	65.70 ± 5.75	69.20 ± 6.93	0.004
Education	12.80 ± 3.69	11.90 ± 3.92	12.80 ± 3.21	0.480
HTN, *n* (%)	18 (38)	22 (44)	6 (50)	0.719
DM, *n* (%)	8 (17)	7 (14)	2 (17)	0.914
Hyperlipidemia, *n* (%)	17 (36)	27 (54)	6 (50)	0.203
Coronary artery disease, *n* (%)	5 (11)	6 (12)	1 (8)	0.930
Depression, *n* (%)	3 (6)	2 (4)	0 (0.0)	0.618
Current smoking, *n* (%)	8 (17)	3 (6)	0 (0.0)	0.093
Current alcohol consumption, *n* (%)	24 (51)	26 (52)	8 (67)	0.610
Laboratory				
SBP	125 ±15.8	124 ±15.8	126 ± 19	0.891^†^
DBP	73.2 ± 7.29	72.7 ± 10.2	71.7 ± 9.62	0.612^†^
FBS	99.6 ± 18.4	104 ± 16.9	101 ± 17.1	0.314^†^
T-chol	187 ± 32.4	187 ± 45.5	173 ± 26.1	0.427^†^
LDL	122 ± 29.4	124 ± 44.2	111 ± 28.4	0.640^†^
HDL	56 ± 13.6	54.5 ± 12.2	53.8 ± 14.7	0.527^†^
TG	126 ± 56.1	123 ± 67.7	120 ± 62.9	0.753^†^
Brain image				
Atrophy, *n* (%)	3 (6.4)	60 (12)	6 (50)	<0.001^†^
MTA scale-Rt	0.57 ± 0.74	0.72 ± 0.76	1.25 ± 0.75	0.017^†^
MTA scale-Lt	0.49 ± 0.72	0.62 ± 0.78	1.25 ± 0.75	0.006^†^
MTA scale-sum	1.06 ± 1.39	1.34 ± 1.44	2.50 ± 1.45	0.005^†^
Hippocampal volume-Rt (mm^3^)	4128 ± 419	4049 ± 426	4024 ± 602	0.341
Hippocampal volume-Lt (mm^3^)	3944 ± 422	3887 ± 425	3701 ± 632	0.376^†^
Hippocampal volume-sum (mm^3^)	8072 ± 802	7936 ± 820	7726 ± 1189	0.196
Cognitive function				
GDS	3.13 ± 3.19	2.3 ± 2.62	3 ± 3.10	0.452
MMSE	28.7 ± 1.29	28.4 ± 1.27	28.6 ± 1.38	0.456^†^
MoCA	26.3 ± 2.48	25.8 ± 2.61	24.5 ± 2.28	0.101^†^
MoCA-memory	2.76 ± 1.88	2.66 ± 1.56	2.08 ± 1.56	0.434^†^
ADAS-Cog	14.3 ± 5.07	15.4 ± 5.47	18.2 ± 6.28	0.156
ADAS-language	0.26 ± 0.49	0.40 ± 0.93	0.42 ± 0.79	0.348^†^
ADAS-memory	11.4 ± 4.23	12.4 ± 4.56	14.5 ± 4.27	0.037
ADAS-praxia	2.51 ± 1.60	2.48 ± 1.59	2.75 ± 1.71	0.937^†^

Key: SBP, systolic blood pressure; DBP, diastolic blood pressure; FBS, fasting blood sugar; T-chol, total cholesterol; LDL, low-density lipoprotein; HDL, high-density lipoprotein; TG, triglyceride; H-EPVS, hippocampal enlarged perivascular space; MTA, medial temporal atrophy; GDS, geriatric depression scale; MMSE, Mini-Mental State Examination; MoCA, Montreal Cognitive Assessment; ADAS-Cog, The Alzheimer's Disease Assessment Scale-Cognitive Subscale

† *Kruskal Wallis test*.

### Degree of H-EPVS and PI and Mean Flow Velocity of the Middle Cerebral Artery and Posterior Cerebral Artery

The degree of H-EPVS was not found to be related to PI and mean flow velocity of the MCA and PCA (data not shown). Although there was a trend toward increased PI values with H-EPVS degree, the *p*-values of the right PCA PI were 0.058 (P1) and 0.084 (P2).

### Risk Factor of Memory Cognitive Function

To study the risk factor associated with memory cognitive function, we divided subjects into two groups by ADAS-Cog memory score (Table 3, score ≥9 vs. score <9). In univariate analysis, ADAS-Cog memory score was associated with age (score ≥9 vs. score <9, 66.00 ± 6.10 vs. 63.00 ± 4.85; *p* = 0.025) and education (score ≥9 vs. score <9, 12.0 ± 3.82 vs. 13.7 ± 3.82; *p* = 0.034). ADAS-Cog memory score was associated with H-EPVS count, MTA scale sum score and hippocampal total volume (score ≥9 vs. score <9, 1.11 ± 1.22 vs. 0.593 ± 0.797; *p* = 0.041; score ≥9 vs. score <9, 1.51 ± 0.690 vs. 0.852 ± 0.949; *p* = 0.042; score ≥9 vs. score <9, 7,878 ± 867 vs. 8,255 ± 775; *p* = 0.047). ADAS-Cog memory score was also associated with degree 2 of H-EPVS and MTA scale sum score ≥4 (*p* = 0.035; *p* = 0.034).

**Table 3:** Demographic, laboratory results, and imaging features of the subjects according to ADAS-Cog memory score.

	**ADAS-Cog memory <9 (*n* = 27)**	**ADAS-Cog memory ≥9 (*n* = 82)**	***P*-value**
Demography			
Male, *n* (%)	12 (44)	45 (55)	0.472
Age	63 ± 4.85	66 ± 6.10	0.025
Education	13.7 ± 3.24	12.0 ± 3.82	0.034^‡^
HTN, *n* (%)	7 (26)	39 (48)	0.080
DM, *n* (%)	2 (7)	15 (18)	0.295
Hyperlipidemia, *n* (%)	13 (48)	37 (45)	0.959
Coronary artery disease, *n* (%)	3 (11)	9 (11)	>0.999
Depression, *n* (%)	1 (4)	4 (5)	>0.999
Current smoking, *n* (%)	5 (19)	6 (7)	0.191
Current alcohol consumption, *n* (%)	13 (48)	45 (55)	0.700
Laboratory			
SBP	123 ± 12.3	125 ± 17.1	0.621
DBP	71.9 ± 8.33	73.1 ± 9.12	0.535
FBS	102 ± 14.3	102 ± 18.6	0.918^‡^
T-chol	189 ± 40.8	184 ± 37.8	0.596
LDL	120 ± 40.7	122 ± 35.7	0.797^‡^
HDL	61.7 ± 14.1	52.9 ± 11.9	0.002
TG	123 ± 41.2	124 ± 67.5	0.934^‡^
H-EPVS			
Degree			0.042
0	16 (59)	31 (38)	
1	11 (41)	39 (47)	
2	0 (0)	12 (15)	
Degree ≥2	0 (0)	12 (15)	0.035
Total count of H-EPVS	0.593 ± 0.797	1.11 ± 1.22	0.041
Hippocampal atrophy			
Atrophy, *n* (%)	0 (0)	15 (18)	0.038
MTA scale-Rt	0.444 ± 0.506	0.805 ± 0.823	0.034
MTA scale-Lt	0.407 ± 0.572	0.707 ± 0.824	0.082
MTA scale sum	0.852 ± 0.949	1.51 ± 0.690	0.042
MTA scale sum ≧ 4	0 (0)	13 (16)	0.034
Hippocampal volume-Rt (mm^3^)	4,236 ± 415	4,029 ± 442	0.035
Hippocampal volume-Lt (mm^3^)	4,019 ± 398	3,849 ± 462	0.054^‡^
Hippocampal volume-sum (mm^3^)	8255 ± 775	7878 ± 867	0.047

Key: SBP, systolic blood pressure; DBP, diastolic blood pressure; FBS, fasting blood sugar; T-chol, total cholesterol; LDL, low-density lipoprotein; HDL, high-density lipoprotein; TG, triglyceride; H-EPVS, hippocampal enlarged perivascular spaces; ADAS-Cog, The Alzheimer's Disease Assessment Scale-Cognitive Subscale; MTA, medial temporal atrophy

‡ *Wilcoxon rank sum test*.

### Independent Contributions on Memory Cognitive Function

**Figure 3:**
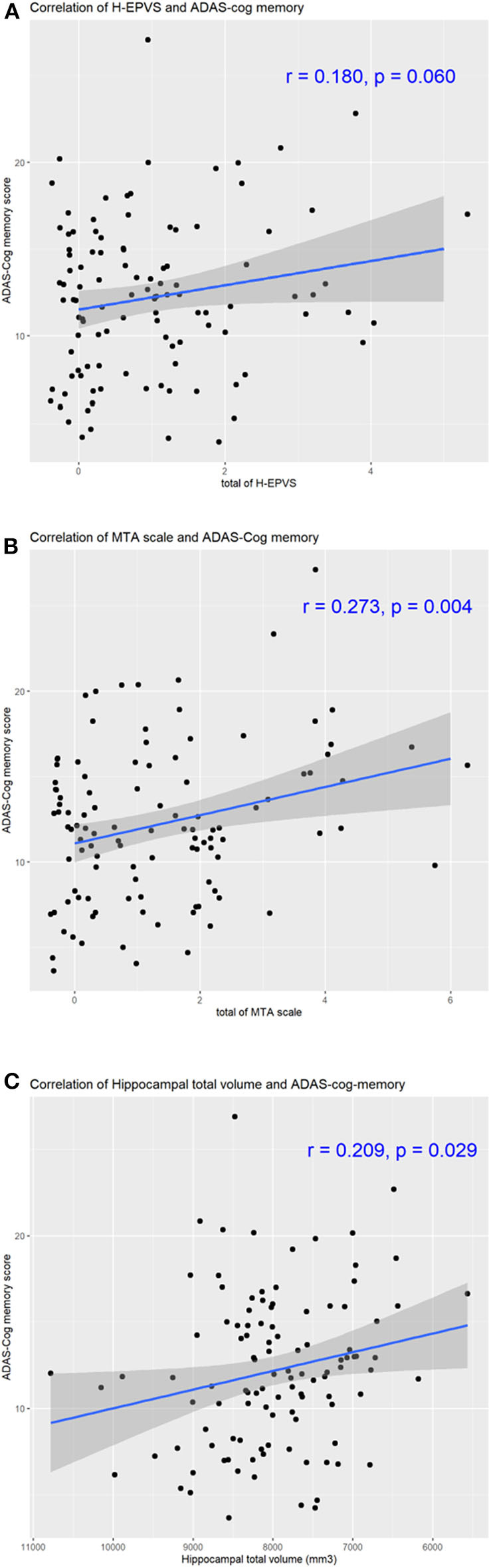
Correlation of H-EPVS, hippocampal atrophy and cognitive function. The line in each graph was obtained from the univariate regression model. R means correlation coefficients. Correlation of H-EPVS and ADAS-Cog memory **(A)**. Correlation of MTA scale and ADAS-Cog memory **(B)**. Correlation of hippocampal total volume and ADAS-Cog memory **(C)**. H-EPVS, hippocampal enlarged perivascular spaces; MTA, medial temporal atrophy; ADAS-Cog, The Alzheimer's Disease Assessment Scale-Cognitive Subscale.

Considering multivariate models to determine predictors of ADAS-Cog memory score, MTA scale score was found to be independently associated with ADAS-Cog memory score [Table 4, MTA scale sum score ≥4, *p*-value 0.011, β (95% CI) 3.467 (0.802–6.133)]. However, after adjustments for age, sex, education, HDL, and hypertension, the degree of H-EPVS and hippocampal total volume were not found related to ADAS-Cog memory score (Table 4, degree 2, *p* = 0.113; *p* = 0.090). With adjustment of multivariable factors, education duration was found to be independently associated with memory cognitive function (Table 4, *p* < 0.05). We showed the correlation of memory cognitive function and H-EPVS count, MTA scale sum score and hippocampal total volume (Figure 3).

**Table 4:** The impact of H-EPVS, MTA and hippocampal volume on ADAS-Cog memory.

	**ADAS-Cog memory**	
	**β (95% CI)**	***p*-value**
**H-EPVS and ADAS-Cog memory**		
**Model 1**		
Degree of H-EPVS ≥2	2.142 (−0.515 to 4.799)	0.113
Age	0.123 (−0.025 to 0.272)	0.101
Sex (male)	1.244 (−0.536 to 3.024)	0.169
Education	−0.314 (−0.547 to −0.082)	0.009
HDL	−0.027 (−0.090 to 0.037)	0.409
HTN	0.292 (−1.447 to 2.031)	0.740
**MTA scale and ADAS-Cog memory**		
**Model 2**		
MTA scale sum ≥4	3.467 (0.802 to 6.133)	0.011
Age	0.086 (−0.065 to 0.236)	0.261
Sex (male)	0.816 (−0.947 to 2.579)	0.361
Education	−0.287 (−0.516 to −0.059)	0.014
HDL	−0.022 (−0.085 to 0.041)	0.490
HTN	0.242 (−1.464 to 1.947)	0.780
**Hippocampal volume and ADAS-Cog memory**		
**Model 3**		
Hippocampal volume	−0.001 (−0.002 to 0.0001)	0.090
Age	0.108 (−0.045 to 0.260)	0.163
Sex (male)	1.722 (−0.169 to 3.613)	0.074
Education	−0.297 (−0.529 to −0.065)	0.013
HDL	−0.027 (−0.091 to 0.036)	0.396
HTN	−0.033 (−1.806 to 1.793)	0.970

*Multivariable linear regression analyses with covariates (Age, Sex, Education, HDL, HTN) were built to assess the association between ADAS-Cog-memory and degree of H-EPVS and hippocampal atrophy. In each model, ADAS-Cog-memory score was the outcome and degree of H-EPVS, MTA sum score and hippocampal volume*.

*Key: H-EPVS, hippocampal enlarged perivascular spaces; HDL, high-density lipoprotein; MTA, medial temporal atrophy score; ADAS-Cog, The Alzheimer's Disease Assessment Scale-Cognitive Subscale*.

## Discussion

In this prospective study of 109 subjects, we found the age plays a key role in the development of H-EPVS in non-dementic elderly population. Besides, memory cognitive function was found to be independently associated with MTA but not with the degree of H-EPVS.

Enlarged perivascular spaces commonly referred to as the Virchow-Robin space, which was originally described by Durand Fardel in 1843, detailed by Rudolf Virchow in 1851 (27), and later confirmed by Charles-Philippe Robin as donut-shaped channels surrounding the brain's blood vessels (28). It is believed to be caused by extensions of the subarachnoid space surrounding the penetrating arteries (29). The compromised fluid and toxin clearance from the brain is a causal role of EPVS. We investigated whether the degree of H-EPVS is related to increased arterial PI and cognitive impairment.

A similar age effect was previously detected in previous studies (3, 4, 30, 31). Moreover, EPVS become increasingly common with aging (32, 33). Hypertension and poor treatment compliance of hypertension were independently related to the large number of H-EPVS (3, 4, 30). However, our study found no relationship between hypertension and degree of H-EPVS. H-EPVS was observed in 56.9% of individuals. This result is similar to those of previous studies (2–4).

The memory cognitive function and association of the degree of H-EPVS are controversial. In previous studies, the degree of H-EPVS does not appear to be associated with the development of dementia or cognitive ability (3, 11, 13). A meta-analysis of five population-based studies reported that higher H-EPVS counts were linked to better cognitive performance in memory (5). We divided subjects into two groups by ADAS-Cog memory score (score ≥9 vs. score <9) to determine the risk factors of memory cognitive function. In multivariate models to determine predictors of ADAS-Cog memory score, MTA scale score were found to be independently associated with ADAS-Cog memory score (MTA scale sum score ≥4, *p* = 0.011). However, the degree of H-EPVS was not found to be related to ADAS-Cog memory score (degree 2, *p* = 0.113). This result means that the risk factor of memory cognitive function is related to MTA but not the degree of H-EPVS.

In our study, we found that the degree of H-EPVS was associated with MTA scale score. We showed the correlation of H-EPVS and MTA scale score, hippocampal total volume (Figure 2, correlation efficient, 0.273; *p* = 0.004; correlation efficient, 0.199; *p* = 0.038). Therefore, we believe that H-EPVS may be a secondary phenomenon of MTA. However, it is not completely clear whether H-EPVS follows the development of clinically overt MTA. Future studies with larger-scale longitudinal designs are warranted to examine whether MTA contributes to H-EPVS. The relationship between EPVS and brain atrophy is controversial. Brain atrophy is an independent risk factor for severe EPVS in the basal ganglia (34, 35). In contrast to our study, other studies reported the association of the degree of H-EPVS and higher volume of the hippocampus. Another study reported that H-EPVS was not influenced by hippocampal atrophy (2). Because H-EPVS was associated with age in our study, there may be a possibility that MTA is associated with H-EPVS even in non-dementic subjects.

With adjustment of multivariable factors, education duration was found to be independently associated with memory cognitive function (*p* < 0.05). In previous studies, more years of education moderate genetic risk and memory function associated with amyloid β by increasing cognitive function capacity (36, 37).

We failed to find any significant association between the degree of H-EPVS and cerebrovascular pulsatility. Shi et al. reported that high PI was only associated with basal ganglia but not centrum semi-ovale PVS (31). They explained that the arterioles in the two locations showed differences in associations of PVS. PI showed difficulty in assessing the resistance of small vessel and reflecting microvascular pathology. Moreover, our study participants have lesser vascular risk factors. Our results showed that the *p*-values of right PCA PI were 0.058 and 0.084, which are more related to the degree of H-EPVS than MCA values. It may be because the origin of hippocampal arteries is the PCA or PCA branches, but further study with large population is needed (38).

The strength of our study is that we performed detailed cognitive tests compared with previously published papers. They used simple cognitive tests, such as MMSE, Isaacs set tests, or trail making tests (3, 5, 13, 30), which are crude measures of cognitive function. However, the ADAS-Cog is more accurate, both in differentiating individuals with normal cognition from those with impaired cognition and in assessing the extent of cognitive impairment in individuals. With the imaging study in healthy non-dementic subjects, we could study the correlation of H-EPVS and cognitive ability with lesser confounding factors, such as white matter hyperintensity, lacunar infarcts, and brain atrophy.

However, our study has several limitations. First, the use of a semiquantitative evaluation for rating the degree of H-EPVS can limit our ability to detect small effects and differences. Extremely small ischemic cavities in the cerebral tissue might have been misclassified as EPVS. Future advances in automatic/semiautomatic computational segmentation algorithms for EPVS quantification may improve cross-validation between studies (39). For hippocampal atrophy, we used the semiquantitative and quantitative analyses. But in multivariate models to determine predictors of ADAS-Cog memory score, results were inconsistent (*p* = 0.011 vs. *p* = 0.090). It is because our participants were non-dementic and smaller group. Hippocampal volume is useful only in distinguishing late onset AD and amnestic-early onset AD from healthy population (40). So, it is difficult to find differences of memory score by hippocampal volume in this group. Third, it is well-known that the hippocampus plays an important role in declarative and episodic memory. ADAS-Cog memory subscales principally evaluate verbal memory. Therefore, it would be more appropriate to use a more complete memory scale with other tests. Fourth, we did not consider the relationship between H-EPVS and other radiologic markers of cognitive impairment, such as total brain volume and cortical thickness. Fifth, our study has a cross-sectional design, which restricts our interpretation of data with respect to cause and consequence. Finally, PI and MFV were not available in 18% of subjects due to poor temporal window. We excluded their data from the analysis, which may result in selection bias in that the excluded individuals may be older, may be less educated, and may carry more vascular risk factors.

## Conclusion

The present findings suggest that age plays a key role in the development of H-EPVS in the non-dementic elderly population. The degree of H-EPVS was found be associated with MTA scale score. However, TCD parameters were not related to the degree of H-EPVS. With adjustment of multivariable factors, memory cognitive function was found to be independently associated with MTA, but not with the degree of H-EPVS.

## Data Availability Statement

The original contributions presented in the study are included in the article/supplementary material, further inquiries can be directed to the corresponding author/s.

## Ethics Statement

The studies involving human participants were reviewed and approved by the Ethical Committee of the Sungkyunkwan University. The patients/participants provided their written informed consent to participate in this study.

## Author Contributions

All authors listed have made a substantial, direct and intellectual contribution to the work, and approved it for publication.

## Conflict of Interest

The authors declare that the research was conducted in the absence of any commercial or financial relationships that could be construed as a potential conflict of interest.
